# Endoscopic Minimally Invasive Beating-Heart Tricuspid Valve Surgery Without Jugular Vein Cannulation or Caval Snaring: Clinical Outcomes Using Novel Percutaneous Venous Cannulation

**DOI:** 10.3390/medicina62071380

**Published:** 2026-07-17

**Authors:** Razan Salem, Orestis Mallis Kyriakides, Feras Kabbesh, Hamid Naraghi, Mohamed Zeriouh, Andreas Däuwel, Bujar Maxhera, Michael Weissenfels, Diyar Saeed

**Affiliations:** 1 Department of Cardiovascular Surgery, Heart Center Niederrhein, Helios Klinikum Krefeld, Health and Medical University Düsseldorf/Krefeld, 40221 Düsseldorf, Germany; razan.salem@helios-gesundheit.de (R.S.);; 2 Department of Humanmedizin, University Witten/Herdecke, 58455 Witten, Germany; 3 Department of Anesthesiology, Helios Klinikum Krefeld, Health and Medical University Düsseldorf/Krefeld, 40221 Düsseldorf, Germany; 4 Department of Cardiovascular Surgery, Clinical Perfusion, Heart Center Niederrhein, Health and Medical University Düsseldorf/Krefeld, 40221 Düsseldorf, Germany

**Keywords:** tricuspid valve repair, minimally invasive cardiac surgery, beating-heart surgery, venous cannulation, air embolism prevention, endoscopic surgery, Smart Cannula

## Abstract

*Background and Objectives*: Endoscopic beating-heart tricuspid valve (TV) surgery is increasingly adopted in cardiac surgery due to its association with improved postoperative recovery. Conventional approaches require jugular vein cannulation to establish adequate bicaval venous drainage, and caval snaring is considered mandatory during beating-heart TV procedures to prevent intraoperative air lock. We report our single-center experience with a novel percutaneously placed venous cannula (Smart Cannula) that enables minimally invasive TV surgery without jugular cannulation or caval snaring. *Materials and Methods*: Between February 2025 and May 2026, 31 consecutive patients underwent endoscopic beating-heart TV surgery using the Smart Cannula system for venous drainage. The device is a stent-like cannula with distributed wall perforations allowing sufficient venous return while preventing air lock. Preoperative atrial fibrillation was present in 21 patients (68%). Six patients (19%) underwent isolated TV procedures; the remainder had concomitant procedures. Mean age was 68.2 ± 10.8 years; 16 patients (52%) were female. Two different cannula lengths were used: 680 mm (*n* = 15) and 730 mm (*n* = 16). Six patients (19%) had history of prior cardiac surgery. *Results*: All procedures were completed without intraoperative air lock. Two patients (6%) required intraoperative conversion to sternotomy. Mean cardiopulmonary bypass time was 163 ± 52 min. Seven patients (23%) underwent beating-heart procedures with no aortic cross-clamping; in 24 patients (77%), aortic cross-clamping was required for concomitant procedures (mean cross-clamp time 79 ± 28 min), with tricuspid repair completed on the reperfused beating heart. Re-exploration for bleeding occurred in two patients (6%). Median ICU stay was 5 days and median hospital stay was 10 days. New permanent pacemaker implantation was required in three patients (10%). Thirty-day mortality was 0%. Postoperative echocardiography at discharge demonstrated TR Grade 0 in 61%, mild TR in 32%, and moderate TR in 3% of patients. *Conclusions*: In this first and largest published series on endoscopic beating-heart TV surgery without caval snaring or jugular vein cannulation, we have shown that the process is feasible and safe, eliminating the need for jugular vein cannulation and caval snaring while maintaining no occurrence of intraoperative air lock. The device enables a simplified venous drainage strategy without compromising operative safety or early clinical outcomes.

## 1. Introduction

Tricuspid valve (TV) disease is a highly prevalent and clinically significant condition, yet historically undertreated. Secondary (functional) tricuspid regurgitation (TR) is the most common form, arising from right ventricular dilatation and annular dilation in the setting of left-sided valve disease, atrial fibrillation, or pulmonary hypertension [1,2]. There is growing evidence that severe TR is associated with substantial morbidity and mortality, and that concomitant correction at the time of left-sided valve surgery improves long-term outcomes [3,4]. Furthermore, isolated TV surgery for residual or recurrent TR after prior procedures has gained increasing acceptance [5].

Minimally invasive approaches to TV surgery, performed through right minithoracotomy and under videoscopic guidance, have gained widespread adoption over the past two decades. These techniques offer shorter recovery times, reduced blood transfusion requirements, and equivalent repair durability compared to conventional sternotomy [6,7]. The beating-heart technique, in which the procedure is performed on the normothermic, perfused heart without aortic cross-clamping, is particularly appealing for isolated TV surgery, as it avoids ischemia–reperfusion injury and obviates the need for cardioplegia [8].

However, beating-heart TV surgery presents a specific technical challenge: the open right atrium and tricuspid annulus create a pathway for air entrainment into the venous drainage system. This can lead to air lock within the cardiopulmonary bypass circuit, causing sudden and dangerous loss of venous return. To prevent this, conventional protocols require placement of an additional jugular venous cannula to enable bicaval cannulation, followed by snaring of both the superior and inferior caval vein by tapes or occlusion ballons [9,10]. This strategy carries the attendant risks of jugular vein cannulation, including access-site complications, potential for carotid puncture, and risk of thrombosis.

**Figure 1:**
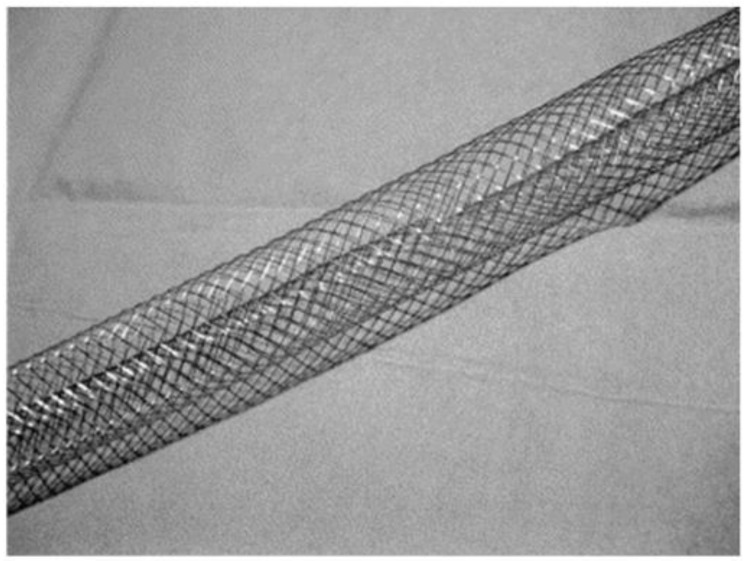
Stent-like virtually wall-less Smart Cannula, (Smartcanula LLC, Zug, Switzerland).

The Smart Cannula (Smartcanula LLC, Zug, Switzerland) is a novel percutaneously deployed venous drainage device designed specifically to address this limitation (Figure 1). The Smart Cannula is a novel stent-like device featuring multiple wall perforations distributed along the whole intracorporal shaft of the cannula. Once positioned, these perforations allow venous drainage from multiple entry sites simultaneously, preventing the formation of a sustained air column within the cannula lumen in the event of air entrainment through the open cardiac chambers, rendering caval snaring unnecessary.

To date, only limited clinical data are available describing the use of this novel cannulation strategy in the context of beating-heart TV surgery. We present our single-center experience with 31 consecutive patients who underwent endoscopic TV surgery using the Smart Cannula for venous drainage, with the primary aim of reporting the feasibility, safety, and early clinical outcomes of this approach.

## 2. Materials and Methods

### 2.1. Study Design and Patient Population

This is a single-center, retrospective observational study of all consecutive patients who underwent endoscopic TV surgery using the Smart Cannula for venous drainage at our institution between February 2025 and May 2026. Patients were identified through the institutional cardiac surgery database. Indications for TV repair were due to functional degenerative TR, secondary to left-sided mitral valve disease, or secondary to atrial fibrillation.

The study was approved by the local ethics committee, and the requirement for individual informed consent was waived given the retrospective nature of the study. All patient data were anonymized prior to analysis. Follow up was complete in all patients.

### 2.2. Surgical Technique

**Figure 2:**
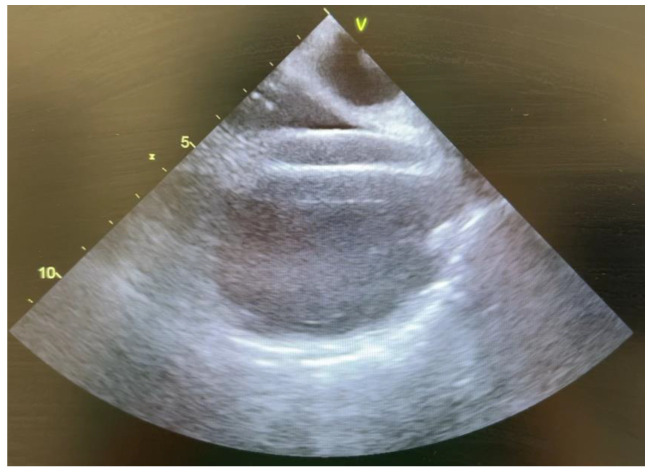
Expanded Smart Cannula in TOE.

All patients were operated on via a right lateral minithoracotomy (4th or 5th intercostal space). Cardiopulmonary bypass (CPB) was established via standard percutaneous ultrasound guided femoral artery and femoral vein cannulation, as previously described [11]. Arterial access was achieved using a Freelife femoral arterial cannula (Eurosets, Medolla, Italy) in most cases. For venous drainage, the Smart Cannula (Smartcanula LLC, Zug, Switzerland) was introduced percutaneously via the femoral vein and advanced into the right atrium and superior vena cava under transoesophageal echocardiographic (TOE) guidance (Figure 2).

Two cannula lengths were used (680, and 730 mm) and were selected based on the patient’s body length and the estimated distance from the femoral vein access point to the superior vena cava. The 680 mm cannula was used for patients with body length of <170 cm, while 730 mm cannula was used for patients ≥ 170 cm.

In patients with isolated TV disease and no concomitant cardiac pathology requiring aortic cross-clamping, the procedure is performed entirely on the beating-heart. Systemic temperature is maintained at 36–37 °C. In all cases, the situs is flooded with CO_2_ via continuous insufflation (at 2.5 L/min) to reduce the risk of residual air. Passive venous line pressure (from the right atrium to the reservoir) amounts to −10 mmHg. Vacuum assistance is necessary in most cases, requiring suction of −30 mbar. Once the right atrium is opened, venous vacuum suction is reduced to −15 mbar. The patient is positioned toward the right side to allow blood to accumulate within the right atrium, away from the tricuspid valve. It is important that the perfusionist adjusts the suction so that the surgical field remains free of blood. During beating heart TV repair, sweep gas flow of the CPB is adjusted from 4 L/min to 8–10 L/min in order to compensate for the CO_2_ rise, which is expected due to the open right atrial chamber and consecutively increased CO_2_ uptake from the insufflated CO_2_ into the bloodstream. In patients with concomitant left-sided valve disease, the mitral valve is addressed first under cardioplegic arrest (Del Nido cardioplegia, antegrade delivery). De-airing maneuvers are performed prior to weaning from CPB. Video S1 shows the CPB line with multiple air bubbles without any event of air lock (Video S1).

Concomitant procedures performed at the same setting included cryoablation for atrial fibrillation, left atrial appendage (LAA) exclusion, and ASD or CABG as indicated.

### 2.3. TV Repair Techniques

Tricuspid annuloplasty was performed using a rigid or semi-rigid ring in all patients with TV repair. The clover technique, as described by Alfieri and colleagues, was applied in selected patients with complex leaflet prolapse or perforation, where a central coaptation stitch was placed to create a double-orifice tricuspid valve [12]. Repair quality was assessed intraoperatively by saline injection testing and confirmed by TOE prior to weaning from bypass.

### 2.4. Data Collection and Definitions

Preoperative data collected included age, sex, body mass index (BMI), TR (MR) grade (graded I–V per current echocardiographic guidelines: mild, moderate, severe, massive and torrential), presence and type of atrial fibrillation, history of prior cardiac surgery, and comorbidities. Operative variables included CPB and aortic cross-clamp times, cannula length, type of procedure, and intraoperative events. Postoperative outcomes included ICU and total hospital length of stay, reintervention for bleeding, reintubation, need for ECMO or continuous veno-venous hemofiltration (CVVH), new pacemaker implantation, cerebrovascular events, respiratory complications, rhythm at discharge, and 30-day mortality. Postoperative echocardiography was performed at discharge in all patients.

Intraoperative air lock was defined as any sudden or sustained loss of venous return attributable to air entrainment causing hemodynamic instability, circuit interruption, or requiring emergent intervention.

### 2.5. Statistical Analysis

Continuous variables are expressed as mean ± standard deviation (SD) for normally distributed data and as median with range or interquartile range (IQR) for skewed distributions. Normality was assessed by inspection of histograms and the Shapiro–Wilk test. Categorical variables are presented as absolute numbers and percentages. Given the descriptive and exploratory nature of this study, no inferential statistical comparisons were performed. All analyses were performed using Python (v3.11) with the pandas and NumPy libraries.

## 3. Results

### 3.1. Patient Characteristics

Between February 2025 and May 2026, 31 patients underwent endoscopic TV surgery using the Smart Cannula at our institution. Baseline patient characteristics are summarized in Table 1. The mean age was 68.2 ± 10.8 years (range 30–91), and 16 patients (52%) were female. Preoperative atrial fibrillation was present in 21 patients (68%), including paroxysmal in 11 (35%), persistent in seven (23%), and permanent AF in three patients (10%). Six patients (19%) had undergone prior cardiac surgery. Predominant preoperative TR was Grade III in 23 patients (74%) and Grade II in eight patients (26%). Concomitant MR was present in 28 patients (90%), most commonly Grade III (*n* = 22, 71%). One patient had a pre-existing pacemaker.

### 3.2. Operative Details

Right lateral minithoracotomy was employed in 29 patients (94%); the remaining two patients (9%) underwent median sternotomy. All Smart Cannula placements were performed via the femoral vein. Cannula length selection was guided by body habitus; the 680 mm cannula was used in 15 patients (48%), and 730 mm in 16 patients (52%). Femoral arterial access was used in all cases.

Six patients (19%) underwent isolated TV procedures. The remaining 25 patients (81%) had concomitant procedures: MV annuloplasty in 21 (68%), MV replacement in four (13%), cryoablation for AF in 17 (55%), LAA exclusion in 14 (45%), ASD closure in one (3%), and single-vessel CABG in one (3%). TV repair was accomplished by annuloplasty alone in 26 patients (84%), while the clover technique in addition to annuloplasty was employed in four patients (13%). One patient underwent isolated TV replacement.

Seven patients (23%) underwent beating-heart left-sided cardiac surgery without aortic cross-clamping. In the remaining 24 patients (77%), an aortic cross-clamp was required for the concomitant cardiac procedure; TV repair was then completed on the reperfused beating heart after cross-clamp release, while the patient remained on CPB. Del Nido cardioplegia was used in all arrested-heart cases, administered in an antegrade fashion. Mean CPB time was 163 ± 52 min (range 58–303 min). Mean aortic cross-clamp time in patients who required arrest was 79 ± 28 min (range 36–126 min).

Operative details are summarized in Table 2.

### 3.3. Intraoperative Events

No intraoperative air lock occurred in any patient. The Smart Cannula provided optimal venous drainage without any events of drainage inadequacy requiring cannula repositioning. Two patients (6%) required intraoperative conversion from minithoracotomy to median sternotomy due to inadequate visualization during a complex redo procedure. Two patients required intraoperative ECMO implantation for hemodynamic instability during weaning from CPB; and one required postoperative ECMO support. All Smart Cannula placements were technically successful without cannula-related complications.

### 3.4. Postoperative Outcomes

Thirty-day mortality was 0%. Postoperative outcomes are detailed in Table 3. The median ICU stay was 5 days (mean 7.2 ± 12.0 days; range 1–67). The prolonged ICU stays in a small number of patients reflected the requirement for prolonged ECMO support. Median hospital stay was 10 days (mean 12.5 ± 11.1 days; range 4–67).

Re-exploration for bleeding was necessary in two patients (6%), and reintubation for respiratory failure was required in two patients (6%). One patient required postoperative CVVH for acute kidney injury. New permanent pacemaker implantation was performed in three patients (10%). Non-surgical complications occurred in three patients (9%): cerebrovascular event in one, nosocomial pneumonia in one and cardiac decompensation in one.

At discharge, sinus rhythm was present in 19 patients (61%), atrial fibrillation in eight patients (26%), and pacemaker-dependent rhythm in four patients (13%), of whom three had received a new permanent pacemaker implantation.

### 3.5. Postoperative Echocardiographic Results

Transthoracic echocardiography at discharge was available in all patients. Tricuspid valve repair was effective, with no residual TR (Grade 0) in 20 patients (64%), mild TR in 10 patients (32%), and moderate TR in one patient (3%). No patient had severe, massive or torrential TR. For patients with concomitant mitral valve procedures, 20 patients (64%), had no residual MR, mild MR in nine patients (29%), and moderate MR in two patients (6%). Right ventricular function was preserved in 26 patients (84%), mildly reduced in four (13%), and moderately reduced in one (3%). Left ventricular function was preserved (>50%) in 25 patients (81%) and mildly reduced (49–41%) in six patients (19%).

## 4. Discussion

In this largest and, to our knowledge, first published study with beating-heart tricuspid valve surgery using novel Smart Cannula in 31 patients, we demonstrate that endoscopic beating-heart TV surgery can be safely performed using the Smart Cannula for venous drainage, without the need for jugular vein cannulation or intraoperative caval snaring. The principal findings are: (1) No incidence of intraoperative air lock. (2) No 30-day mortality. (3) Excellent early echocardiographic results with no TR or mild TR in 97% of patients at discharge. (4) A low rate of major complications, consistent with published outcomes for minimally invasive TV surgery.

Beating-heart TV surgery confers specific physiological advantages over arrested-heart approaches. By preserving continuous myocardial perfusion and avoiding ischemia–reperfusion injury, the technique may reduce the risk of right ventricular dysfunction in the postoperative period—a feared complication given the right ventricle’s particular sensitivity to ischemic insult and volume overload [13]. Several series have demonstrated the feasibility of beating-heart TV repair, particularly for isolated or concomitant procedures where the anticipated surgical complexity of the mitral valve does not preclude this strategy [8,14]. However, conventional beating-heart TV surgery universally requires establishment of a separate superior venous cannulation line, most commonly via the internal jugular vein, to enable adequate bicaval venous drainage and facilitate caval snaring during intracardiac manipulation [9,10]. This additional access step introduces potential complications including jugular vein hematoma, thrombosis, or inadvertent carotid puncture, and adds procedural complexity, particularly in patients who have undergone prior sternotomy or radiation therapy to the neck.

We truly believe that this approach is particularly advantageous in patients with history of previous cardiac procedures. Avoiding adhesions and diminishing the need to dissect around the caval veins substantially facilitates this otherwise high-risk procedure as it reduces the risk of injury and bleeding, usually resulting in an emergency re-sternotomy. In our cohort, we have included six patients (19%) with prior cardiac surgery, in whom we were able to show an excellent result using this technique.

The Smart Cannula addresses this limitation through a fundamentally different mechanism of venous drainage. The device’s distributed perforations along its whole intravascular segment allow venous blood to be aspirated from multiple levels simultaneously. When air enters the right atrium through the open surgical field, the distributed inflow ports allow continued drainage from adjacent portions of the cannula that remain blood-filled, thereby preventing the formation of a sustained air column that would otherwise interrupt venous return. This design effectively decouples the anti-air-lock function from the requirement for mechanical caval occlusion, enabling safe beating-heart intracardiac surgery without jugular cannulation. In our cohort, this mechanism proved entirely reliable across 31 consecutive procedures encompassing a range of surgical complexities and concomitant interventions.

Our results are consistent with the only previously published research employing this device in TV surgery. The cannula was initially described for use in minimally invasive mitral valve surgery, where it provided effective venous drainage via the femoral route without jugular supplementation [15]. The adaptation to beating-heart TV procedures represents a logical extension of this technology. Our series is, to our knowledge, the largest cohort to date specifically reporting Smart Cannula use in the context of beating-heart TV repair. To date, only a tutorial video demonstrating the use of Smart Cannula in beating-heart TV surgery has been published on MMCTS [16], and there have been no studies released regarding the clinical outcomes of this procedure.

The echocardiographic outcomes in our series compare favorably with published data. In large series of minimally invasive TV repair, rates of TR Grade 0–I at discharge range from approximately 80–85% [6,17,18]. Our rate of 97% (no TR in 61%, mild TR in 32%) reflects both effective repair technique and the selection of patients with predominantly functional TR amenable to annuloplasty. The use of the clover technique in four patients with complex pathology demonstrates the versatility of the approach even in anatomically challenging cases.

The rate of new permanent pacemaker implantation (10%) in our cohort is consistent with published ranges for TV surgery. Reported rates of new postoperative complete heart block and pacemaker dependency following TV repair vary from 5% to 20% in contemporary series, reflecting proximity of the repair to the atrioventricular node and the His bundle [19]. The high burden of preoperative AF in our cohort (68%) is also noteworthy; cryoablation was performed concomitantly in 55% of patients, consistent with current guidelines recommending rhythm surgery in patients with persistent or long-standing persistent AF undergoing valvular procedures [20]. Among patients who received cryoablation, 47% were discharged in sinus rhythm.

The conversion rate (6%) and re-exploration for bleeding rate (6%) in our series are within expected ranges for minimally invasive redo and complex cardiac surgery. Two patients required intraoperative ECMO implantation; both cases involved severely reduced preoperative right ventricular function and complex concomitant procedures and were not attributed to the venous cannulation strategy. The zero 30-day mortality despite a clinically complex cohort (19% redo, 68% pre-existing AF, mean age 68 years) is encouraging.

This study has several important limitations. First, the retrospective single-center design introduces selection bias; patients were selected for this approach by their operating surgeons, and unmeasured confounders may have influenced outcomes. Second, the sample size is modest at 31 patients, limiting the statistical power of any comparative analyses. Still, this is the largest published series on this approach. Third, follow-up was limited to hospital discharge; durability of TV repair and long-term freedom from TR recurrence are not reported. However, our study focuses on feasibility of using this approach and not on long term results of the performed procedures. Fourth, the echocardiographic core was performed at discharge without blinded core-laboratory assessment, and inter-observer variability in TR grading is recognized. Fifth, the study period spans a learning curve with a novel device. Prospective multicenter studies with longer follow-up are required to validate these findings and to formally compare the Smart Cannula approach with conventional bicaval cannulation strategies.

## 5. Conclusions

Endoscopic beating-heart TV surgery without caval snaring or jugular vein cannulation using the Smart Cannula for femoral venous drainage is feasible and safe, eliminating the need for jugular vein cannulation and caval snaring without compromising venous drainage adequacy or operative safety. In this series, no intraoperative air lock events occurred, no 30-day mortality event was reported, and echocardiographic repair quality was excellent at discharge. The Smart Cannula represents a promising simplification of the venous cannulation strategy for minimally invasive TV surgery, with potential benefits in patient safety, procedural efficiency, and applicability to redo cases. Larger prospective studies with longer follow-up are warranted.

## Figures and Tables

**Table 1:** Baseline patient characteristics.

Characteristic	Value (*n* = 31)
Age, years, mean ± SD (range)	68.2 ± 10.8 (30–91)
Female sex, *n* (%)	16 (52)
BMI, kg/m^2^, mean ± SD	26.8 ± 4.5
Preoperative atrial fibrillation, *n* (%)	21 (68)
Paroxysmal	11 (35)
Persistent/Permanent	7 (23)/3 (10)
Pulmonary Hypertension	
Mild (sPAP 35 mmHg–44 mmHg)	6 (19)
Moderate to severe (sPAP > 45 mmHg)	7 (22)
Redo cardiac surgery, *n* (%)	6 (19)
Preoperative TR grade, *n* (%)	
Moderate	8 (26)
Severe	23 (74)
Preoperative MR, *n* (%)	
None	3 (10)
Moderate	2 (6)
Moderate-severe	22 (71)
Severe	1 (3)
Combined/other	2 (6)
Left ventricular dysfunction	2 (6)
HFmrEF 41–49%	
Pre-existing pacemaker/ICD, *n* (%)	1 (3)
Chronic kidney disease (eGFR < 60 mL/min/1.73 m^2^)	10 (32)
Congestive Hepatopathy (moderately elevated liver enzymes)	3 (10)

BMI = body mass index; sPAP = systolic pulmonary artery pressure; TR = tricuspid regurgitation; MR = mitral regurgitation; HFmrEF = Heart Failure with mildly reduced Ejection Fraction; eGFR = estimated glomerular filtration rate.

**Table 2:** Intraoperative characteristics.

Variable	Value (*n* = 31)	95% CI
Surgical access		
Right minithoracotomy, *n* (%)	29 (94)	
Median sternotomy, *n* (%)	2 (6)	
Smart Cannula length, *n* (%)		
680 mm	15 (48)	
730 mm	16 (52)	
Arterial cannulation site	Femoral (100%)	
Isolated tricuspid procedure, *n* (%)	6 (19)	
Concomitant procedures, *n* (%)		
MV annuloplasty	21 (68)	
MV replacement	4 (13)	
TV repair—annuloplasty only	26 (84)	
TV repair—annuloplasty + clover technique	4 (13)	
Left atrial Cryoablation	17 (55)	
LAA exclusion	14 (45)	
ASD closure	1 (3)	
CABG	1 (3)	
Cardioplegic arrest (Del Nido), *n* (%)	24 (77)	
Beating-heart left-sided cardiac surgery (no cross-clamp), *n* (%)	7 (23)	
CPB time, min, mean ± SD (range)	163 ± 52 (58–303)	
Aortic cross-clamp time, min, mean ± SD (range)	79 ± 28 (36–126) [*n* = 24]	
Intraoperative air lock, *n*	0	
Conversion to sternotomy, *n* (%)	2 (6)	0.8–21.4%
Intraoperative ECMO implantation, *n* (%)	2 (6)	0.8–21.4%

CI = confidence interval; CPB = cardiopulmonary bypass; MV = mitral valve; TV = tricuspid valve; LAA = left atrial appendage; ASD = atrial septal defect; CABG = coronary artery bypass grafting; ECMO = extracorporeal membrane oxygenation.

**Table 3:** Postoperative outcomes.

Outcome	Value (*n* = 31)	95% CI
30-day mortality, *n* (%)	0 (0)	0.0–11.2%
ICU stay, days, median (mean ± SD)	5 (7.2 ± 12.0)	
Hospital stay, days, median (mean ± SD)	10 (12.5 ± 11.1)	
Re-exploration for bleeding, *n* (%)	2 (6)	0.8–21.4%
Postoperative ECMO, *n* (%)	1 (3)	0.1–16.7%
Renal replacement therapy (CVVH), *n* (%)	1 (3)	
New permanent pacemaker implantation, *n* (%)	3 (10)	2.0–25.8%
Cerebrovascular event, *n* (%)	1 (3)	0.1–16.7%
Nosocomial pneumonia, *n* (%)	1 (3)	
Cardiac decompensation, *n* (%)	1 (3)	
Discharge rhythm		
Sinus rhythm, *n* (%)	19 (61)	
Atrial fibrillation, *n* (%)	78(26)	
Pacemaker-dependent rhythm, *n* (%)	4 (13)	
Postoperative echocardiography at discharge		
TR Grade 0 (none), *n* (%)	20 (64)	
TR mild, *n* (%)	10 (32)	
TR moderate, *n* (%)	1 (3)	
MR Grade 0 (none), *n* (%)	20 (64)	
MR mild, *n* (%)	9 (29)	
MR moderate, *n* (%)	2 (6)	
Preserved RV function, TAPSE > 16, *n* (%)	26 (84)	
Preserved LV function, EF > 50, *n* (%)	25 (81)	

CI = confidence interval; ICU = intensive care unit; ECMO = extracorporeal membrane oxygenation; CVVH = continuous veno-venous hemofiltration; TR = tricuspid regurgitation; MR = mitral regurgitation; RV = right ventricle; LV = left ventricle; EF = ejection fraction.

## Data Availability

The data presented in this study are available on request from the corresponding author due to privacy reasons.

## Supplementary Materials

The following supporting information can be downloaded at: https://www.mdpi.com/article/10.3390/medicina62071380/s1.
